# Literature Review on Needs of Upper Limb Prosthesis Users

**DOI:** 10.3389/fnins.2016.00209

**Published:** 2016-05-12

**Authors:** Francesca Cordella, Anna Lisa Ciancio, Rinaldo Sacchetti, Angelo Davalli, Andrea Giovanni Cutti, Eugenio Guglielmelli, Loredana Zollo

**Affiliations:** ^1^Unit of Biomedical Robotics and Biomicrosystems, Università Campus Bio-Medico di RomaRome, Italy; ^2^Italian Workers' Compensation Authority (INAIL), Vigorso di BudrioBologna, Italy

**Keywords:** upper limb prosthesis, patient needs, grasping, prosthesis requirements, PNS-based prosthesis

## Abstract

The loss of one hand can significantly affect the level of autonomy and the capability of performing daily living, working and social activities. The current prosthetic solutions contribute in a poor way to overcome these problems due to limitations in the interfaces adopted for controlling the prosthesis and to the lack of force or tactile feedback, thus limiting hand grasp capabilities. This paper presents a literature review on needs analysis of upper limb prosthesis users, and points out the main critical aspects of the current prosthetic solutions, in terms of users satisfaction and activities of daily living they would like to perform with the prosthetic device. The ultimate goal is to provide design inputs in the prosthetic field and, contemporary, increase user satisfaction rates and reduce device abandonment. A list of requirements for upper limb prostheses is proposed, grounded on the performed analysis on user needs. It wants to (i) provide guidelines for improving the level of acceptability and usefulness of the prosthesis, by accounting for hand functional and technical aspects; (ii) propose a control architecture of PNS-based prosthetic systems able to satisfy the analyzed user wishes; (iii) provide hints for improving the quality of the methods (e.g., questionnaires) adopted for understanding the user satisfaction with their prostheses.

## Introduction

The human hand is a powerful tool for sensing and operating in the environment, as well as a very sophisticated means for physical and social interaction. It allows the human beings to accomplish sophisticated movements, from power to precision tasks, thanks to the large number of Degrees of Freedom (21 DoFs for the hand and 6 for the wrist) and the paramount role played by thumb opposition.

Voluntary motor commands accounts for a large amount of proprioceptive and exteroceptive information and are translated into neural and muscular activity to actuate the limb, thanks to the skeletal structure. The hand is very important for social interaction and establishes the frontiers between what belongs to the Self and what belongs to the environment. Hand loss can be perceived as a devastating damage since it affects the level of autonomy, limiting the capability of performing working, social, and daily living activities (ADLs). Ultimately, it changes people lifestyle.

**Figure 1:**
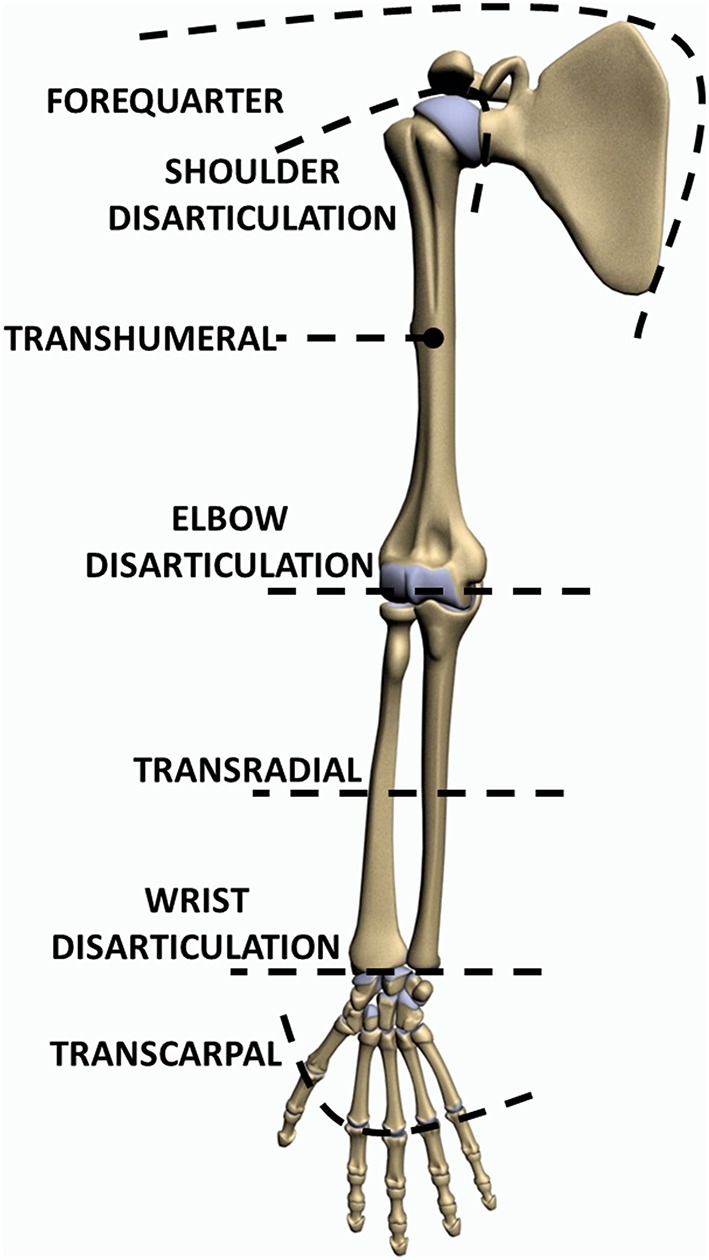
**Levels of upper limb absence**.

The levels of upper limb loss can be classified as transcarpal, wrist disarticulation, transradial, elbow disarticulation, transhumeral, shoulder disarticulation and forequarter (Figure 1).

**Figure 2:**
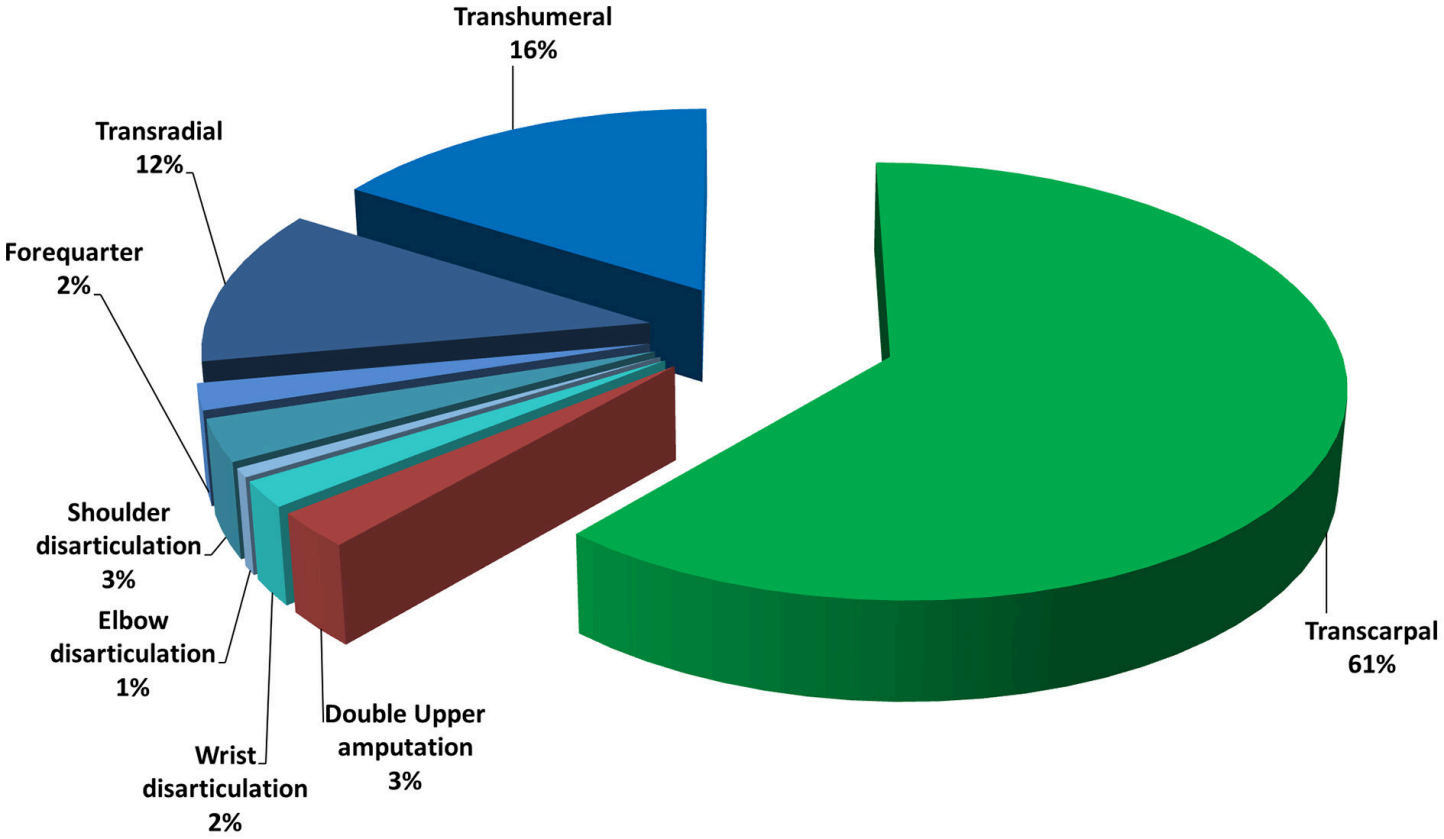
**Statistics on level of upper limb absence in Italy and United Kingdom**.

Around 541,000 Americans suffered from different levels of upper limb loss in 2005; the number of cases is expected to double at least by 2050 (Ziegler-Graham et al., 2008). Approximately 3500 and 5200 upper limb amputations are reported each year in Italy^1^ and in UK, respectively. The incidence of the different levels of upper limb loss is also shown in Figure 2: 16% trans-humeral, 12% transradial, 2% forequarter, 3% shoulder disarticulation, 1% elbow disarticulation, 2% wrist disarticulation, 61% transcarpal, and 3% bilateral limb loss. Traumatism is the first cause of upper limb amputation, predominantly for males^2^. It is followed by neoplasia and vascular or infectious diseases (Frontera and Silver, 2014).

The loss of a limb interrupts the closed-loop with the brain that takes place by means of the efferent and afferent pathways, responsible for motor control and sensory feedback, respectively.

Upper limb prostheses (Fumero and Costantino, 2001) can be classified into two main categories on the basis of their functioning: passive prostheses (which in turn are divided into cosmetic and functional) and active prostheses (which include body-powered and externally powered).

Cosmetic prostheses mainly aim at the aesthetic substitution of the missing body part, while functional prostheses have the purpose of facilitating very specific activities, such as those related to work or sport.

Within the active prostheses, the body-powered ones are controlled by cables fastened to the sound limb of the amputee by means of harnesses. The high expenditure of energy required to the user is one of the drawbacks of this kind of prostheses. Instead, externally powered prostheses exploit an external power source to raise the energy needed for movement, for example a battery pack. They can be further classified into two sub-categories: myoelectric (which are controlled through electromyographic (EMG) signals) and electric (ideal for example for phocomelic people who can command the prosthesis by means of external buttons).

Despite the advances in technology over the last 50 years, today's upper limb prostheses are still affected by relevant limitations. One of the main engineering challenges in the development of prosthetic devices is, on one hand, to embed actuators, sensors and electronic components into a prosthesis of the same size and weight of the replaced hand or limb, on the other hand, to improve prosthesis control that notably affects functionality.

Intuitive control can be developed by extracting the user's intention from signals recorded in a non-invasive way [through surface EMG electrodes (sEMG; Dohnalek et al., 2013), ultrasound imaging (Gonzales and Castellini, 2013), force myography (FMG; Wininger et al., 2008)] or else in an invasive way [by means of Implantable Myoelectric Sensors (IMES; Pasquina et al., 2015), neural interfaces (Dhillon et al., 2004)] from the Peripheral or Central Nervous System. Invasive surgical procedures, such as Targeted Muscle Reinnervation (TMR), have been applied in some cases of very proximal limb loss to allow an intuitive prosthesis control through myoelectric interfaces.

Several researches have been carried out in this field, but a lot of effort is still required for improving the interfaces. Desired requirements are: (i) real-time, direct, robust and simultaneous control of multiple DoFs in a natural and intuitive manner, (ii) bidirectional communication with the Peripheral Nervous System (PNS), and (iii) fast learning. In most of the existing control strategies, limited information (i.e., shoulder movements or recorded EMG signals) is used for activating several DoFs of the artificial limb, thus making non-intuitive and unnatural prosthesis control and requiring a huge cognitive load. Classical myoelectric control is based on on/off (Scott and Parker, 1988) or proportional techniques (Fougner et al., 2012). Notwithstanding their wide adoption in the commercially available systems as well as in the clinical practice (Jiang and Farina, 2014), they do not allow the user to simultaneously control more than one DoF (far from the multifunctional control of the natural hand). Moreover, they require a long training phase and suffer from signal degradation due to sweating or inadequate positioning of the socket.

In order to overcome these limitations, several approaches have been proposed, such as ultrasound imaging (Gonzales and Castellini, 2013), FMG (Wininger et al., 2008), TMR (Hijjawi et al., 2006; Miller et al., 2008), pattern recognition techniques (Cloutier and Yang, 2013) applied to EMG signals acquired through implantable (IMES; Pasquina et al., 2015) or surface electrodes (Dohnalek et al., 2013), and neural signals acquired through implantable neural interfaces (Dhillon et al., 2004; Polasek et al., 2009; Rossini et al., 2010).

Most of the aforementioned solutions are still developed in the research field (e.g., ultrasound imaging, neural interfaces, IMES, pattern recognition) without clinical application. Pattern recognition offers the advantage of enabling the simultaneous, independent control of multiple DoFs (Ortiz-Catalan et al., 2014). Nonetheless, performance is affected by arm posture modification, electrode positioning, fatigue, inherent cross talk in the surface signal and displacement of the muscles during contraction. Pattern recognition also requires very long learning sessions; further, its performance in the real context is different from the laboratory settings (Jiang and Farina, 2014), thus limiting its clinical applicability and acceptance. In January 2015 the first device based on pattern recognition and surface electrodes (COAPT^3^) has been commercialized.

The adoption of IMES has been tested on transradial amputees (Pasquina et al., 2015) to provide intuitive and stable myoelectric control. Promising preliminary results have been obtained (i.e., the prosthesis control is more robust to limb position and environmental conditions with respect to the use of superficial electrodes), but IMES cannot be employed when the sensing sites are very close each other, or else if the target muscle is small or thin.

Ultrasound imaging has a comparable accuracy with sEMG, but it has lower wearability and is much more sensitive to arm/hand displacement. Further efforts could make this solution a valuable alternative to sEMG (Castellini et al., 2014), especially working on signal processing, component miniaturization, force decoding (Gonzales and Castellini, 2013).

Force myography (Wininger et al., 2008) consists in placing force sensing resistors (FSRs) on the limb surface and detecting the volumetric changes of the residuum. It can provide information about the user's motion intention (Li et al., 2012) and grip force (Wininger et al., 2008), and does not depend on a precise placement of the sensors. However, preliminary results show that grasp classification accuracy is still lower than EMG (Cho et al., 2016).

Natural control occurring through the PNS can be achieved by means of peripheral neural interfaces (Navarro et al., 2005). The main limitations of intraneural electrodes are related to the high invasiveness and signal degeneration due to fibrotic reaction; additionally, the computational burden for signal processing and classification on the efferent pathway is considerably higher than for EMG (Cloutier and Yang, 2013). On the other hand, it is really effective in returning tactile perception on the afferent pathway (Raspopovic et al., 2014).

As for neural interfaces, invasiveness is a drawback also for TMR and IMES. In Engdahl et al. (2015) it was shown that the surgical risk is the main concern of individuals with upper limb loss about using invasive techniques for prosthesis control. An online questionnaire proposed to 104 American people outlined that, notwithstanding the preference for non-invasive interfaces (83% was interested in EMG control), invasive techniques can be accepted when a higher level of functionality is offered (63% expressed interest in TMR and 68% in neural interfaces).

**Figure 3:**
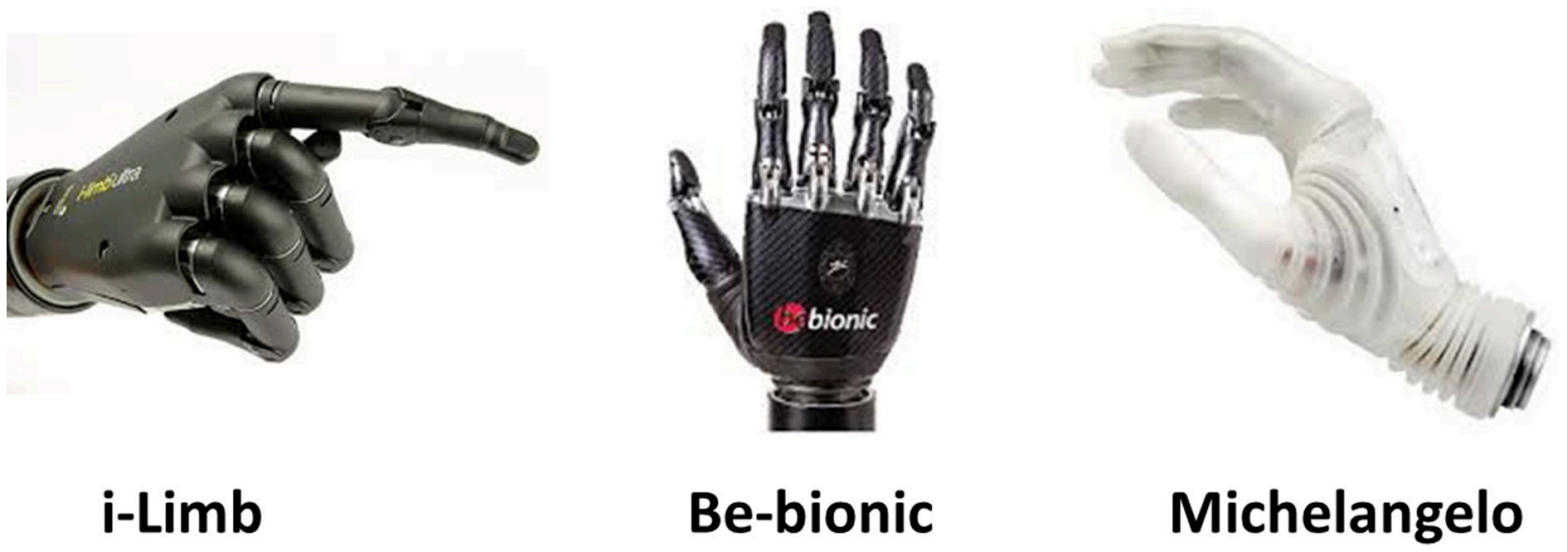
**Most advanced commercially available prosthetic hands**.

While control of upper limb prostheses is still coarse, progress in mechanics (Atzori and Muller, 2015) is notable, as confirmed by the advanced poliarticulated myoelectric prosthetic hands available on the market (i.e., the i-Limb^4^, the Bebionic^5^, and the Michelangelo^6^ hands in Figure 3; Belter et al., 2013). They enable several grasping tasks thanks to the number of DoFs, as shown in Table 1.

**Table 1:** **Characteristics of poliarticulated commercially available prosthetic hands (Belter et al., 2013)**.

**Hand and company name**	**i-Limb by touch bionics**	**Bebionic by RSL steeper**	**Michelangelo by ottobock**
Weight	443–515 g	550–598 g	420 g
No of actuators	6 DC motors	5 DC motors	2 DC motors
No of DoFs	6	6	2
Active DoFs	F/E of MCP joint of each finger and thumb opposition	F/E of MCP joint of each finger	F/E of all the fingers contemporarily and thumb opposition
Passive DoFs	–	Thumb opposition (i.e., it is changed by the user)	–
Joint coupling mechanism	Tendon linking MCP to PIP	Linkage spanning MCP to PIP	Cam design with links to all fingers
Grasping configuration	Power, precision, lateral, hook, finger-point	Power, precision, lateral, hook, finger-point	Opposition, lateral, neutral mode
Maximum applied force	100–136 N	140 N	70 N

One of the main limitations of current devices is represented by the lack of an intuitive and reliable interface able to map the user motion volition to real movement of the prosthesis (Dhillon and Horch, 2005). Finally, the necessity of an extensive training required to properly manage the artificial hand, the lack of sensory feedback and the noise produced by the actuators during movements make the prosthetic hands still far from fully addressing the users' needs (Clement et al., 2011), see Section Needs Analysis of Upper Limb Prosthesis Users. Therefore, this paper intends to perform an updated literature review on the analysis of user needs; the ultimate goal is to provide valid indications on the requirements that prostheses should have to increase user acceptability. In Section Aim of the Study, the study purposes will be explained in detail.

The paper is structured as follows: Section Aim of the Study presents the aim of this study; Section Method describes the method followed for performing this review; Section Needs Analysis of Upper Limb Prosthesis Users provides an overview of the studies on user needs, while Section Discussion reports the requirements to be satisfied by the prosthetic system in order to meet the patient's needs. Finally, Section 6 offers critical considerations and suggests future pathways of development.

## Aim of the study

This paper intends to carry out an in-depth study of the literature on needs analysis of upper limb prosthesis users in order to consolidate current knowledge in the field and delineate the users' priorities as well as main requirements for upper limb prostheses. Although individual solutions (in terms of mechatronic design, socket and control) could be proposed for each single user in order to meet the patient's specific needs, general insights can be provided for improving user satisfaction, thus paving the way to subsequent user-tailored solutions.

This work has the twofold purpose of (i) helping focus research efforts toward the development of prostheses that are functional and appealing for individuals with upper limb loss, by addressing their needs; (ii) providing foundations for future studies to more in-depth explore user needs and priorities that inevitably can change with technology advancements and market development.

The expected added value provided by this work is to complete the current knowledge on the users' needs with more recent papers, by critically evaluating and comparing (when possible) the available results, and pointing out inconsistencies and neglected aspects. Hence, an updated list of user requirements and prosthesis features has been built and is available for the research community. The ultimate goal is to provide indications for the development of future prosthetic technologies that are appealing to individuals with upper limb loss.

## Methods

A wide literature search updated to March 2016 has been performed from the following databases: PubMed, Google Scholar, Cochrane Database of Systematic Reviews. The keywords (and their combinations) adopted for the research are the following: upper limb, prosthesis, amputation level, upper limb loss, myoelectric, body-powered, cosmetic, prosthesis control, user needs, satisfaction, priorities, abandonment, sensory feedback. All publications in English and Italian languages appeared between 1980 and 2016 have been considered. Additional articles extracted from the references of the papers identified in the electronic search have been included. The inclusion criteria for selecting the publications relevant for the review purpose are the following: studies on upper limb prosthesis users that account for cosmetics, functionality and comfort; studies on upper limb prosthesis users who wear cosmetic, body-powered and myoelectric prostheses; studies reporting user opinions about desired features, design priorities and activities of daily living they desire to perform with the prosthesis.

**Figure 4:**
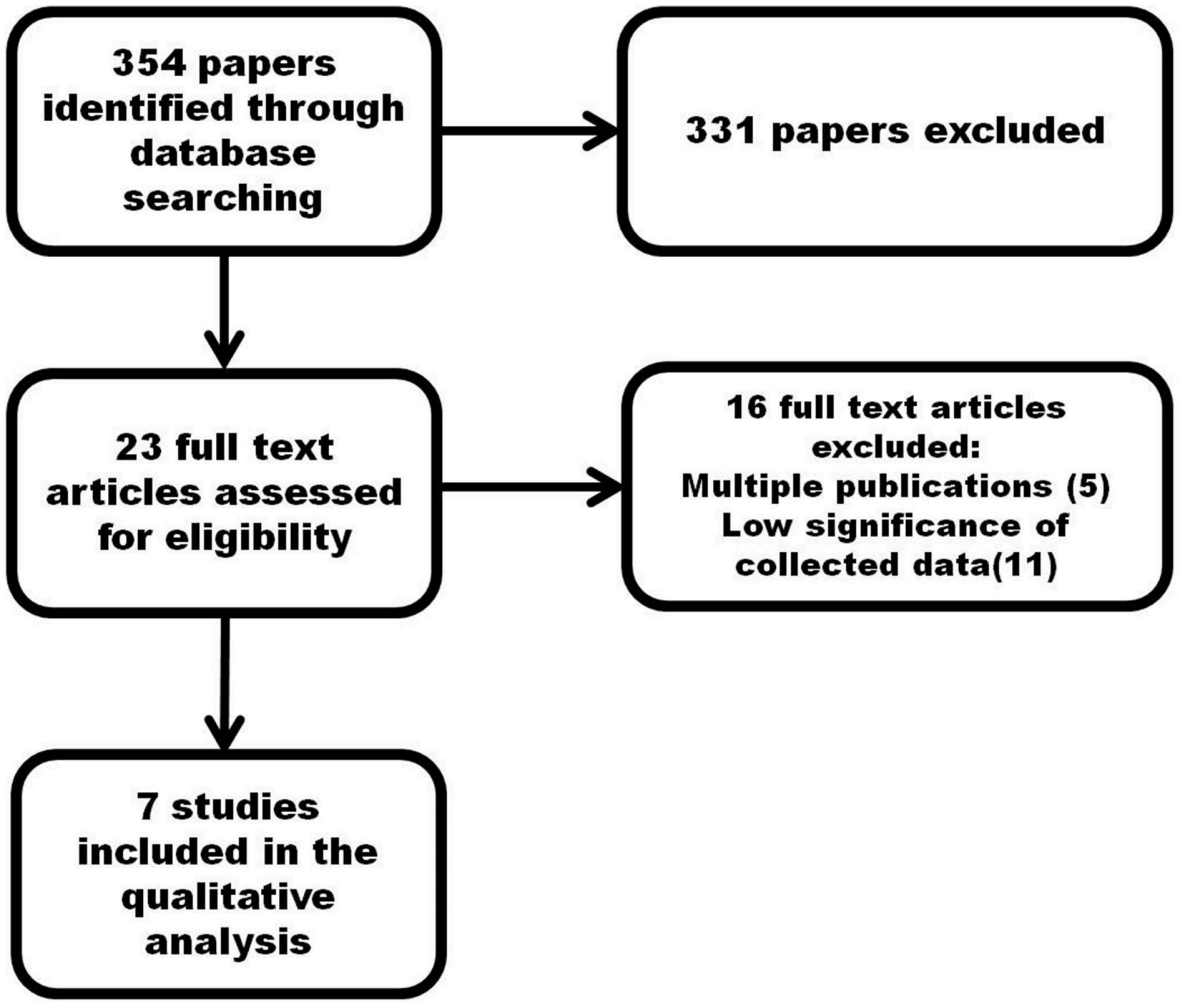
**Flowchart of the search and inclusion process**.

A flowchart of the search and inclusion process is shown in Figure 4. A total of 354 papers has been gathered by using the aforementioned search method. The abstracts matching the inclusion criteria have been selected. When appropriate, the full paper has been read. Therefore, from the initial 354 papers, 331 have been excluded since they did not meet the inclusion criteria. The remaining 23 papers have been carefully read. Sixteen of these have been excluded from the following discussion since already reviewed several times in the literature or because the reported data were not significant for the purposes of this work. The last seven studies will be discussed in the following. They also include the results of previous studies that are not explicitly mentioned in this review.

The selected studies adopted different methods and tools, and provide heterogeneous data that are difficult to be analyzed in a systematic way. However, despite the difficulty to find a primary outcome measure, interesting common features have been extracted and a list of factors necessary to assess user demands on prostheses has been identified.

## Needs analysis of upper limb prosthesis users

The main issues faced by the literature over the years are related to: the type of activities that the prostheses can help perform (Van Lunteren et al., 1983) or that could be performed with a prosthesis (Fraser, 1998); the explanation of why several amputees prefer not to use the prosthesis (Fraser, 1993; Kejlaa, 1993; Biddiss and Chau, 2007; Peerdeman et al., 2011); the identification of the problems related to job before and after amputation (Heger et al., 1985; Wright et al., 1995). However, the studies often use different methods of analysis and adopt different selection criteria of the involved users; moreover, they are also limited to a single site or country. In the attempt of performing a comprehensive analysis of the users' needs, the selected papers were in-depth analyzed and differences and similarities were reported, aiming at identifying general requirements for several users.

The seven selected relevant studies are Kyberd and Hill (2007), Biddiss et al. (2007), Jang et al. (2011), Pylatiuk et al. (2007), Østlie et al. (2012), Bouffard et al. (2012), and Lucchetti et al. (2015). Special attention has been dedicated to the comparative analysis of user experience with myoelectric, electric, body-powered and passive upper limb prostheses, independently of the level of limb loss. In all the studies, questionnaires were used to collect data from upper limb prosthesis users. In Lucchetti et al. (2015), a questionnaire was used for physiological assessment, and practical tests were adopted for assessing functional abilities. In Østlie et al. (2012), questionnaires, clinical tests and interviews were used. Three studies, i.e., Biddiss et al. (2007), Jang et al. (2011), and Pylatiuk et al. (2007), involved both adults and children, while the others Kyberd and Hill (2007), Østlie et al. (2012), Bouffard et al. (2012), and Lucchetti et al. (2015) were focused only on adults.

The number of subjects involved in the experimental groups ranged from 6 in Lucchetti et al. (2015) to 307 in Jang et al. (2011) (for a total of *n* = 958 users). All the seven analyzed studies are focused on the analysis of myoelectric prosthetic users, while four of them report priorities and satisfaction levels of body-powered prosthesis users (Biddiss et al., 2007; Jang et al., 2011; Østlie et al., 2012; Bouffard et al., 2012), and four studies are concerned with cosmetic prosthesis users (Kyberd and Hill, 2007; Biddiss et al., 2007; Jang et al., 2011; Østlie et al., 2012).

The participants were asked to answer questionnaires about functional, aesthetical and commercial aspects of the prostheses.

The studies are structured as follows and details are reported in Table 2:

- In Kyberd and Hill (2007) a study performed in Sweden and UK on 156 patients with unilateral upper limb prostheses is reported. The analysis involved users with different levels of limb loss using myoelectric and cosmetic prostheses. A low percentage of users wore other types of prostheses, but the authors did not provide data regarding their answers.
- In Biddiss et al. (2007), 242 users with different levels of limb loss were asked to provide a list of priorities of prosthetic hand features, sorted by relevance. The authors did not account for the type of limb loss in the data analysis. However, it was observed that individuals with above elbow or bilateral amputation preferred body-powered hooks, while adults with congenital limb loss chose cosmetic hands.
- In Jang et al. (2011) the results from 307 Korean subjects were reported, including 34 bilateral amputees. The analysis involved users with different levels of amputation mainly using cosmetic devices.
- In Pylatiuk et al. (2007), the satisfaction of myoelectric hand users with different levels of amputation has been evaluated by means of an internet survey. 54 German users (34 males, 9 females and 11 children) answered questions about (i) general information regarding the amputation (such as date, reason, level, used prosthesis); (ii) time of use of the prosthesis; (iii) perceived noise, weight and appearance of the prosthesis; (iv) activities where the prosthesis is used and requirements for future improvements.
- In Østlie et al. (2012), the perceived usefulness of the prosthesis and the skills of prosthesis users in ADL tasks were evaluated by means of questionnaires, clinical tests and interviews. Two forty four Norway users compiled questionnaires with the objective of identifying the “pattern of prosthesis wear” (i.e., type and wearing times of prosthetic devices) and assessing the prosthesis usefulness. Fifty users were involved in clinical tests and interviews on 59 tasks, with the aim to evaluate user skills while performing ADLs.
- In Bouffard et al. (2012), 12 people from Quebec with an amputation proximal to the wrist were interviewed. The main purpose of the work was to investigate the influence of the phantom limb pain on the prosthesis use. The satisfaction of the users with their prosthesis was also analyzed.
- In Lucchetti et al. (2015), the results of practical tests and questionnaires on the functional and psychosocial impact of a transradial myoelectric prosthesis was reported. The study involved 6 Italian subjects who compared experience and performance achieved with two different prosthetic hands (i.e., the Michelangelo hand and a traditional tridigital myoelectric prosthetic hand by Otto Bock©).

**Table 2:** **Summary of the reported analysis**.

**Study**	**No. of Subjects**	**Age (years)**	**Type of prosthesis**	**Level of limb loss**	**Questions**	**Answers**	**% of responses**
Kyberd and Hill, 2007	156	>16 years	60% C, 27% Myo, 13% other	58% Tr, 31% Th, 7% Sd	Consumer design priorities	More natural appearance (C, Tr, Th, Sd) Improvements in movement and grip functions (E, Tr, Th, Sd)	37% 70%
Biddiss et al., 2007	242	60% Adults (43 years ± 15) 40% Children (9.5 years ± 6)	BP, C, E	54% Tr, 21% Th, 7% Sd, 16% Wd, 15% Bi	Consumer design priorities	Comfort (C), Function (BP), Comfort (E)—ALL type of limb loss.	35% (C), 29% (BP), 45% (E)
					ADLs the subjects would like to perform	Household maintenance Cooking, eating, dressing, personal hygiene, typing—ALL type of prosthetis	40% 19%
Jang et al., 2011	307	Total mean age: 66 years	80.2% C, 1% Myo, 79.2% other	6.6% Sd, 20.5% Th, 48.4% Tr, 6.6 Wd, 17.9 % Tc, 11% Bi	Consumer design priorities ADLs the subjects would like to perform Cooking, eating, dressing, personal hygiene, typing	Cosmesis and comfort	–^†^ –^†^
Pylatiuk et al., 2007	54	79,6% Adults (30.3 years) 20.4% Children (6.9 years)	Myo	76.9% Tr, 14.8% Th, 5.5% not specified	Consumer design priorities ADLs the subjects would like to perform	Sensory feedback Using cutlery	98% 50%
Østlie et al., 2012	224	Total mean age: 54.7 years	19.9% C, 34.2% Myo, 29.8% BP, 16,1% other	85% Tr, 15% Th	ADLs the subjects would like to perform	Cooking, eating, dressing, personal hygiene	–^†^
Bouffard et al., 2012	12	Total mean age: 56 years	7% BP, 8% Myo, 25% both	71.2% Tr, 28.8% Sd, Th, 4% Bi	ADLs the subjects would like to perform	Eating, personal hygiene, employment and recreation	–^†^
Lucchetti et al., 2015	6	Total mean age: 47 years	Myo	Tr	Consumer design priorities ADLs the subjects would like to perform	Functionality Eating and dressing	–^†^ –^†^

*Tr, Transradial; Th, Trashumeral; Sd, Shoulder disarticulation; Tc, Transcarpal; Bi, Bilateral; Wd, Wrist disarticulation; BP, Body-powered; E, Electric; C, Cosmetic; Myo, Myoelectric*.

*ALL = the answer is independent on the type of limb loss and on the type of prosthesis*.

† *Not reported value*.

Before going in-depth in the results of the aforementioned studies, it is worth observing that the evaluation of usefulness and patient satisfaction for different types of prostheses is notably influenced by the unilateral or else bilateral loss (Davalli and Sacchetti, 2009, in Italian). In case of unilateral patients, the prosthetic device is mostly an aid for the sound limbs, while in case of subjects with bilateral limb loss the prosthesis is the main way to interact with the environment.

This aspect has not adequately been faced in the analyzed studies. In fact, the percentage of the respondents with a bilateral limb loss was typically low: 15% in Biddiss et al. (2007), 11% in Jang et al. (2011), 4% in Østlie et al. (2012), for a total of 77 bilateral amputees. In Kyberd and Hill (2007), Bouffard et al. (2012), and Lucchetti et al. (2015) only unilateral upper limb prosthesis users were considered, while in Pylatiuk et al. (2007) the side of limb loss is not specified. The low percentage with respect to the total number of participants led the authors to report only global results, without distinguishing between unilateral and bilateral prosthetic users. Only in Jang et al. (2011) considerations about prosthesis users with bilateral upper limb loss have been presented. They regard the level of satisfaction, the wearing time, the most difficult ADLs and the occupational status. Over 34 bilateral amputees, 26.5% were “dissatisfied,” 38.2% were “moderately satisfied” and 35.3% were “satisfied” with the currently used prostheses. The usage time of the prosthesis was 8–16 h a day for 67.6% of subjects, 4–8 h for 11.8%, and less than 4 h for 20.6%. The most difficult ADLs resulted to be: tying shoe laces (82.4%), buttoning shirts (79.4%) using scissors (76.5%), opening and drinking a bottle beverage (67.6%) and washing face (58.8%). None of the bilateral prosthesis users continued the same work after the amputation: 14.7% were employed on different tasks, while 85.3% did not return to work.

The study in the literature involving the highest number (i.e., 117) of subjects with bilateral upper limb loss is (Atkins et al., 1996). The results of this study have not been explicitly analyzed in this paper, being an old study already accounted for in the more recent papers discussed here. Nevertheless, it is worth reporting the interesting result that users with bilateral amputation aspired to handle efficiently a large variety of objects, apply high forces and finely control motion thanks to the presence of force sensory feedback (Heckathorne, 1994; Atkins et al., 1996).

Two topics faced in the selected studies are the cost and the maintenance of the prosthesis. Users with body-powered prostheses believe that the cost of their prosthesis is adequate and that the cost for glove replacement is a primary concern; on the other hand, users of myoelectrical prosthesis consider too high the cost of the device and indicate the replacement of gloves and battery as the main maintenance issues. This is also supported by Blough et al. (2010), where the cost analysis on 5-year show that emerging high technology devices will inevitably lead to increasing costs. The cost factors are also related to the different reimbursements policies applied by insurance companies and public healthcare systems.

A comparative analysis of these studies has pointed out three important issues for users wearing body-powered prostheses: (i) the important role of wrist movements; (ii) the need of reducing the visual attention during task execution; (iii) the possibility of coordinating joint movements.

Conversely, users wearing myoelectric prostheses wished to reduce the visual attention and have flexing fingers, thumb opposition to each finger.

The most frequently asked questions are related to (i) general information regarding the limb loss (such as date, reason, level, used prosthesis); (ii) period of time during which the prosthesis is worn during the day; (iii) degree of satisfaction of the prosthesis characteristics, such as aesthetics, weight, comfort, sweating, wrist movements; (iv) level of satisfaction of the prosthesis functional capabilities; (v) activities that the users desire to perform with their prosthesis.

In particular, in Kyberd and Hill (2007) it has been shown that many participants wore the prosthesis at least 8 h a day and used it mainly in activities such as working, driving and sports. Similar results have been shown in Pylatiuk et al. (2007) and Østlie et al. (2012), where the users declared to use the prosthesis for at least 8 h a day especially during work, and in Bouffard et al. (2012), where 75% of the users wore their prosthesis for more than 6 h a day. On the other hand, in Jang et al. (2011) less than half of the amputees (44.7%) used their cosmetic prostheses for at least 8 h a day.

With respect to the degree of satisfaction of the prosthesis characteristics, the importance of different design characteristics for users with different prostheses (passive, body-powered, myoelectric) and the importance of the functional role for active and passive prostheses are reported in Biddiss et al. (2007) (Tables 3, 4, respectively). The ranking of importance is obtained on the basis of the Friedman's Rank Test and multiple answers were proposed in the questionnaire.

**Table 3:** **Design priorities of passive, body-powered and myoelectric prosthesis (Biddiss et al., 2007)**.

**Type of prostheses**	**Design priorities**
Passive	Comfort (2.00)^a^
	Appearance (2.46)
	Function (3.06)
	Durability (3.31)
	Cost (4.18)
Body-powered	Function (2.07)
	Comfort (2.07)
	Durability (3.25)
	Cost (3.73)
	Appearance (3.89)
Myoelectric	Comfort (1.91)
	Function (2.39)
	Appearance (3.01)
	Durability (3.23)
	Cost (4.45)

a *The ranking of importance is reported in parentheses*.

**Table 4:** **Consumer specified importance of functional roles for passive and active prostheses (Biddiss et al., 2007)**.

**Type of prostheses**	**Functional priorities**
Passive	Gripping (2.42)^a^
	Steadying (2.52)
	Manipulating (2.80)
	Appearance (3. 41)
	Body language (4.11)
Active	Appearance (2.35)
	Steadying (2.55)
	Manipulating (3.28)
	Gripping (3.38)
	Body language (3.44)

a *The ranking of importance is reported in parentheses*.

In Biddiss et al. (2007) design priorities for future improvements for children and adults were concerned with weight, overall appearance and comfort (Table 5). This was also confirmed by Pylatiuk et al. (2007), where 77% of respondents judged the prosthesis too heavy. No information about the level of limb loss was reported. In Pylatiuk et al. (2007) as well as in Østlie et al. (2012) the cosmetic appearance obtained the higher degree of satisfaction (50 and 82.2%, respectively).

**Table 5:** **Consumer design priorities emerged from the open-ended question (Biddiss et al., 2007)**.

	**Type of prostheses**		
	**Passive**	**Electric**	**Body-powered**
Design priorities	Weight	Weight	Comfort of harness
	Fit	Glove durability	Weight
	Appearance	Cost	Cost
	Heat	Sensory feedback	Wrist movement/control
	Cost	Fine motor skills/dexterity	Grip strength
	Color	Heat	Reliability
	Appearance under clothing	Appearance	Heat
	Glove durability	Reliability	Sensory feedback
	Control of opening/closing	Independently moving fingers	Ability to maneuver in awkward positions
		Fit	Donning/doffing
		Wrist movement/control	Physical effort needed to use

The prosthesis functional capabilities were analyzed in Kyberd and Hill (2007), where a number of limitations were reported by the subjects notwithstanding the high level of user satisfaction [mean value 6.8 in the range 1 (very dissatisfied) to 10 (very satisfied)]. They mainly regard the impossibility of the prosthesis to adapt the grasping configuration to the object shape, the need for higher precision (as also reported in Biddiss et al., 2007), and the difficulty to wear gloves. Table 6 reports the features identified by the users as the most important to be improved. Often, the same improvements are required for electric and cosmetic prostheses, although with different level of importance. For instance, the features related to movement and grip functions have greater importance for myoelectric prosthesis users rather than for cosmetic prosthesis users. In Pylatiuk et al. (2007) the noise produced by the prosthesis (evaluated too high by 25% of respondents) and the prosthesis grasping speed (considered too slow by 80% of respondents) are two aspects to be improved. These complaints are confirmed in Jang et al. (2011), where only 30% of respondents reported satisfaction with the prosthesis.

**Table 6:** **Prioritization of the features to improve for prosthetic users (Kyberd and Hill, 2007)**.

**Type of prostheses**	**Desired features**
Cosmetic	1) More natural appearance (size, color, surface materials) 2) Control of temperature/transpiration 3) Better glove material 4) Ability to prevent object slipping 5) Increase the wrist RoM 6) Less visual attention 7) Less weight
Myoelectric	1) Ability to move separately the fingers and the thumb 2) Ability to prevent object slipping 3) Adaptability of grip strength 4) Increase the wrist RoM 5) Increase movement speed 6) More natural appearance (size, color, surface materials) 7) Control of temperature/transpiration 8) Reliable precision 9) Less weight 10) Reduction of noise 11) Increase sensory feedback

In the selected studies, many challenging or desired activities have been identified. In particular, in Biddiss et al. (2007), these activities are related to household maintenance, heavy lifting, sports, activities of daily living (i.e., cooking, eating, dressing, personal hygiene, typing) and hobbies (i.e., playing a musical instrument). The most desired activity is using cutlery, followed by handicrafts, personal hygiene, opening and closing a door, dressing and undressing; the least wanted activity is writing with the prosthesis. Furthermore, most users (98%) desire to feel the force applied by the prosthesis during grasping, followed by feeling the temperature and performing additional movements, such as pointing the index finger, moving individual fingers, performing wrist flexion/extension, decreasing visual attention and improving cosmetic glove material. These results are confirmed by Jang et al. (2011) and Lucchetti et al. (2015). In fact, the analysis has pointed out that user priorities in terms of ADLs and grasps are eating, dressing and personal hygiene. Additional fundamental features requested by the users are functionality, cosmetics, improvement of the gloves, wrist motion, reliability, noise, dimension and weight.

The answers allowed us to extract from each reviewed study the consumer design priorities and the ADLs the subjects would like to perform (listed in Table 2).

In Table 2 type of prosthesis, level of absence, population, answered questions and percentage of response have been reported for each study. The percentages of unilateral and bilateral limb loss, of people wearing the different types of prosthesis and of different levels of limb loss are also listed in the table, when available.

The table allows the extraction of some general considerations on the users' needs, which are summarized in the following.

- As shown in Kyberd and Hill (2007), it is worth observing that similar interests and needs have been reported by European and American participants.
- When considered, the level of limb loss did not produce significant differences in the desired features or activities to be performed. However, it is worth remarking that the analyzed studies do not investigate the effect on the expressed needs of the type of limb loss (i.e., unilateral vs. bilateral), which is expected to produce significant differences, as reported in Section Discussion.
- The low level of satisfaction for comfort, appearance and functionality is quite common among the users, in particular for cosmetic and body-powered prostheses. This is confirmed by the percentage of prosthesis use abandonment (20–30%), especially due to the lack of functionality, comfort, appearance and sensory feedback (Biddiss and Chau, 2007). The satisfaction level as well as the prosthesis choice could also depend on cultural, social and working factors. In fact, body-powered prostheses, being characterized by moderate cost, durability and reliability, are preferred by workers who are in labor-intensive manual and outdoor occupations. They do not frequently use myoelectric prostheses (preferred by office workers) since, compared to body-powered prostheses, they are more susceptible to be damaged during hard work, present slower response time and require a long lasting learning phase.
- As reported in Biddiss et al. (2007), body-powered prostheses are preferred in USA, while in Europe cosmetic and myoelectric prostheses are mostly adopted. It could also depend on public health care resources, different in each country.
- According to Kyberd and Hill (2007) and Biddiss et al. (2007) it is possible to conclude that user priorities vary depending on the type of prosthesis. For instance, cosmetic users priorities are prosthesis appearance and comfort; on the other hand, myoelectric and body-powered users pay more attention to function and comfort. Improvements in movement and grip functions are primary requirements for myoelectric prosthesis users. Several studies pointed out the wish to control the grip strength and avoid object slippage.
- Myoelectric prostheses are the most efficient in terms of dexterity and interface intuitiveness (compared to cosmetic and body-powered ones), thanks to the higher functionality and the reduced expenditure of energy required to the user. However, myoelectric users complain about the lack of sensory feedback, which is indicated as the most desired design priority (ranked with the highest score in Pylatiuk et al., 2007).

The analyzed studies lack of a common evaluation criteria of the user satisfaction regarding prosthetic usefulness. In fact, in Kyberd and Hill (2007) satisfaction has been rated on a 5-point scale and in a 10-point scale, respectively, ranging from “very satisfied” (5 or 10) to “very dissatisfied” (1); in Jang et al. (2011) the evaluation scale consisted of subjective judgments (i.e., very satisfied, quite satisfied and very unsatisfied), in Biddiss et al. (2007), Pylatiuk et al. (2007), Østlie et al. (2012), and Jang et al. (2011), the Likert scale and a priority score value have been adopted; the study in Bouffard et al. (2012) adopts the rating used in the questionnaire for Quebec user evaluation of satisfaction with assistive technology. This absence of uniformity makes difficult to extract objective data, allows us to infer only qualitative information. The introduction of practical tests (i.e., SouthHampton Hand Assessment Procedure, Box and Block, etc.), as in Lucchetti et al. (2015), could be useful to perform an objective functional assessment. Analogously in Lindner et al. (2010) measures providing reliable information on the device functionality have been identified.

The obtained data certainly stress the aspect that future research and developments should be consumer-driven.

## Discussion

The performed analysis shows that there is a significant variability in the population of upper limb prosthesis users, due to individual anatomy, lifestyle, etc. Therefore, individual solutions should always be considered for the type of prosthesis, the socket, the control and the training. Nevertheless, needs and requirements in common among the users can be found. In particular, the analysis of the literature on the user needs and priorities allows the definition of the following list of user requirements:

- Performing activities of daily living mainly related to eating and dressing, such as combining fork and knife motion, cutting the meat, handling buttons, tying shoelaces. This is desired by all the users, independently either of the level of limb loss (e.g., transradial, transhumeral) or the type of prosthesis (e.g., body-powered, myoelectric). Therefore, a prosthetic system is required to perform basic grasping actions (corresponding to power, pinch, lateral, neutral and pointing of the index finger) and simple manipulation tasks enabling the execution of ADLs.
- Providing the user with sensory feedback. This requirement is shared by all the users, independently of the type of prostheses and level of limb loss (Tables 3, 6).
- Reliability of hand battery and electrodes for active prostheses users (Tables 5, 6).
- Ability to perform activities with higher strength. The priority for myoelectric and body-powered prosthesis users of controlling the grasping force and applying high level forces is reported in Tables 5, 6.
- Performing actions in a more coordinated manner and with less visual attention. These are skills desired by all the users (Table 6).
- Need to wear prostheses with a high level of anthropomorphism (in terms of size, weight, shape, and color). It is a priority for cosmetic prosthesis users; however, also active prosthesis users are interested in anthropomorphism, as shown in Tables 3, 5, 6.
- Performing stable grasps, thus avoiding slippage. As evident from Tables 4–6, this is desired by all the users. The use of slip sensors and embedded control might be used to face this issue.
- Changing position and orientation of the grasped object, thus entailing capabilities of object manipulation (Tables 3, 5). This is a priority for active prosthesis users.
- Ability to move each finger independently, as in free manipulation. As evident in Tables 5, 6, this is required by myoelectric prosthesis users.
- Improving the performance of thumb, index and middle finger (Table 6).
- Improving precision and efficient handling of small objects. The study in Biddiss et al. (2007) pointed out the need for improved dexterity and fine motor skills.
- Improvement of heat dissipation (Tables 5, 6). This is wished by all the users.
- Reduction of the active prosthesis motor noise (Table 6).
- Prosthetic wrist with at least one degree of passive flexion/extension, given the fundamental role of the wrist in ADLs (Tables 5, 6).
- Improving the variety of gloves (flexible, resistant, hardly getting dirty), making them compatible with very common activities, such as the use of touch screens (Lucchetti et al., 2015).
- Providing the prosthesis with an open hand configuration close to the natural one (Lucchetti et al., 2015).
- Improving device duration (estimated around 150,000 cycle/year; Lucchetti et al., 2015).

It is interesting to note that some of the requirements emerging from the analyzed studies were already present in a 20-years-old paper (Atkins et al., 1996). This represents one of the largest investigations about upper limb amputees (involving 1575 subjects) and a fundamental study on priorities of users wearing body-powered and myoelectric prostheses. Several issues in the Atkins's paper are still supported by recent papers, such as the feeling about the prosthesis costs and the demand for reducing visual attention, increasing the wrist RoM and independently moving the fingers.

Additionally, the performed literature review has allowed us to identify the functional tasks required by the prosthesis users. They are basic movements, e.g.,:

- grasping configurations: lateral, pinch, hook, spherical, cylindrical grasp, flattened hand and the centralized grip
- neutral position: complete hand opening, with a natural configuration
- manipulation movements: rotation, slipping, translation, pointing index and pushing coin,

that can be combined to generate the complex ADLs that the patient desire to perform with the prostheses, such as eating (i.e., using knife and fork, cutting the meat, drinking from a glass), dressing (i.e., tie shoelaces, handling of clothes), type writing, handling cell phone, and opening a door (Biddiss et al., 2007; Kyberd and Hill, 2007; Cloutier and Yang, 2013).

The possibility of executing the grasping and manipulation operations listed above also depends on the kinematic and mechanical features of the poliarticulated prostheses. To this purpose, some basic requirements for the hands can be inferred from the user needs analysis: at least two DoFs for the thumb (the former for opposition, which may also be passive, and the latter for flexion/extension), one DoF for index flexion/extension and one DoF for flexion/extension of the remaining fingers, in the hypothesis of finger underactuated mechanisms. Although such requirements and configurations can be satisfied by most of the commercially available poliarticulated prosthetic hands (as shown in Section Introduction) and hand prototypes, a number of limitations still remain concerning the level of dexterity and grasp capability. This is mainly due to control strategies that are not able to manage and properly combine basic hand movements to generate the desired complex motion.

In order to improve the prosthesis acceptability, a high level of anthropomorphism is also required. It can be addressed by replicating finger number and degrees of freedom, as well as size and weight of the natural hand.

**Figure 5:**
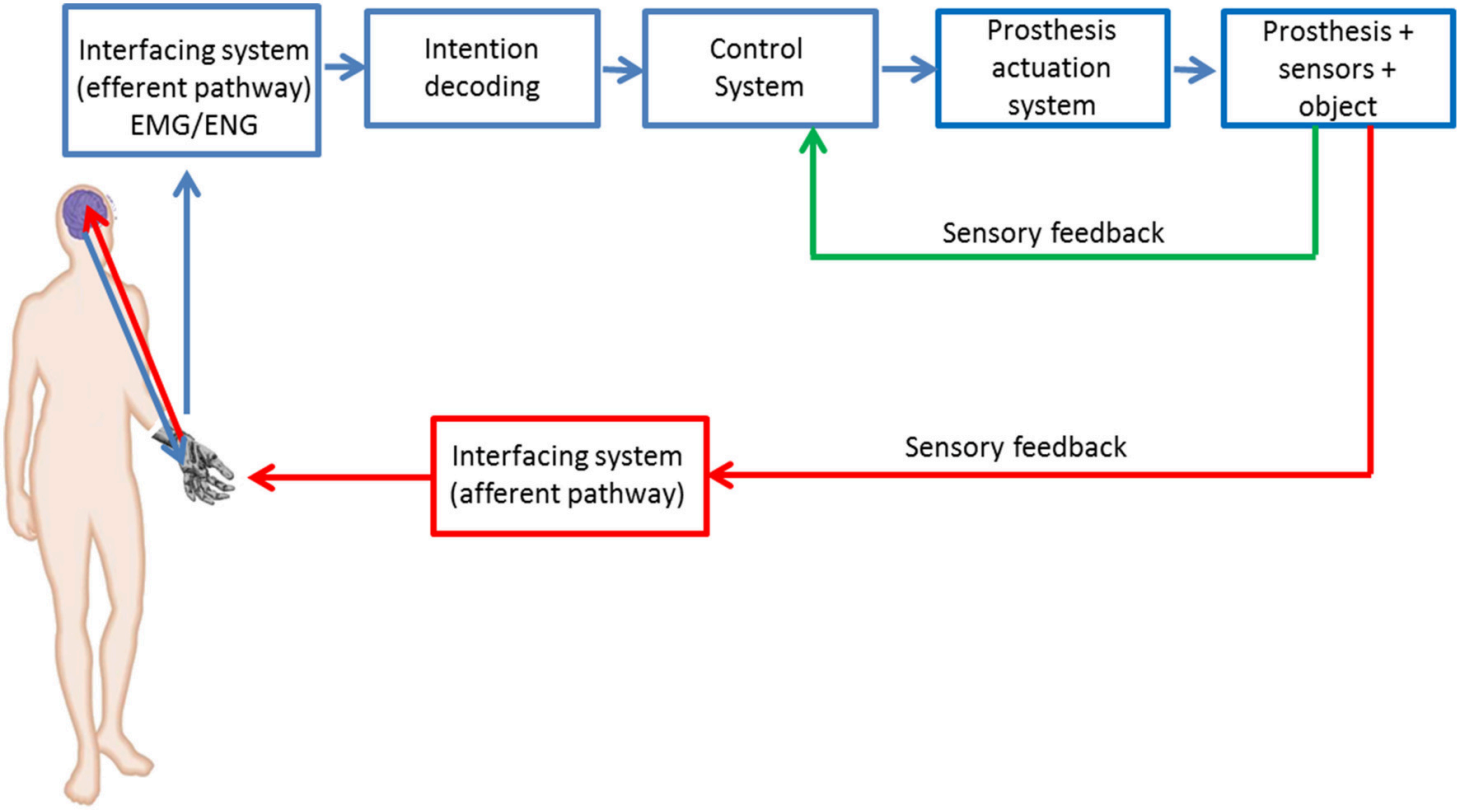
**Block scheme of the PNS-based control of a prosthetic system**.

In Figure 5 an attempt of functional scheme of prosthetic system able to satisfy the wishes emerged from the user needs analysis is proposed. It closes the user in the control loop by establishing a bidirectional communication between the user and the prosthetic system and is composed of the following subsystems (1) the interface responsible for the bidirectional communication with the PNS; (2) the control system driving the prosthesis actuators on the basis of proprioceptive and tactile/force sensory information; (3) the sensory system that returns the tactile feedback about the contact with the manipulated object to the prosthesis embedded control as well as to the amputee, through the bidirectional interface.

The interface with the PNS relies on the acquisition of EMG or ENG signals. In fact, the possibility to perform grasping configurations with low fatigue and more natural and intuitive control makes the EMG-based interfacing system the preferred one as regards the efferent pathway (Weaver et al., 1988). More recently, also ENG signals (Schultz and Kuiken, 2011) have been taken into account for controlling a prosthesis, but this approach is still at research level.

The control system drives the prosthesis actuators starting from the amputee intentions of movement decoded by means of the muscular or neural interfacing system (Schultz and Kuiken, 2011). In order to meet the patient requirements on prosthesis control (in terms of applied force, slippage prevention and movement execution), the control subsystem will receive in input both the patient intention (from EMG/ENG) and the data from a sensory system embedded into the prosthesis.

The needs analysis of upper limb prosthesis also reveals the necessity to provide the prosthetic hand with tactile sensorization for the twofold purpose of performing a force control with the prosthesis and returning force/tactile sensation to the user on the afferent pathway.

Over the years different techniques have been proposed for eliciting tactile sensations (Antfolk et al., 2013; Schofield et al., 2014) by means of invasive or non-invasive interfaces.

Skin stimulation through mechanical vibration, i.e., vibrotactile feedback, is the most used non-invasive technique to provide tactile information during grasping tasks with prostheses, because of the small size and weight (Schofield et al., 2014). The electrotactile sensory substitution consists in stimulating the skin by means of local electrical current. Despite the reduced power consumption and the faster response with respect to vibrotactile technique, the electrotactile feedback does not allow perceiving isolated sensations in a specific task. Prosthesis users often manifest adaptation to vibrotactile and electrotactile stimulation. Moreover, both techniques may cause problems in the integration with myoelectric prostheses by contaminating the myoelectric control signals (Schofield et al., 2014). Mechanotactile feedback makes use of tactors to provide a pressure on the skin. Studies in able-bodied subjects demonstrated the possibility to provide various graduated and discriminated levels of force. Although the mechanotactile feedback has higher spatial discrimination than vibrotactile feedback, the systems necessary to furnish pressure on the skin are typically heavier and larger than other feedback methods.

The electrical stimulation of the peripheral nerve, i.e., neural stimulation, offers the advantage of providing a more natural feedback on the afferent pathway, and potentially recovering the bidirectional communication between the prosthesis and the amputee PNS, using neural electrodes (e.g., cuff or intraneural). Despite the invasiveness, this technique allows selectively eliciting various tactile sensations. In recent studies (Rossini et al., 2010; Ortiz-Catalan et al., 2014; Raspopovic et al., 2014; Tan et al., 2014) it has been demonstrated the possibility to neurally elicit tactile sensations, for example of pressure or vibration, on the subjects involved in the studies. In particular, in Rossini et al. (2010) the possibility of controlling a prosthetic hand by means of efferent neural signals acquired with intraneural electrodes has been demonstrated; in Raspopovic et al. (2014), a first evidence of bidirectional control (intended as efferent and afferent paths closed on the patient) of a prosthetic hand has been developed: the hand control has been performed via EMG sensors and the sensory feedback has been elicited by means of invasive neural interfaces. Electrical stimulation of the nerves for eliciting tactile sensations has also been investigated in Ortiz-Catalan et al. (2014). Cuff electrodes have been used in Tan et al. (2014) with the aim of demonstrating that perceived sensations and perceived areas can be modulated by changing pulses parameters also in chronic implants (i.e., for long period of time).

This paper has provided a literature review on needs and priorities of prostheses users in order to identify the requirements of a prosthetic system. The performed analysis makes it also possible to identify a list of factors necessary to assess user demands on prostheses:

- demographic/personal factors: gender, education level, marital status, ethnicity, occupational status before and after amputation, hobbies/sports;
- limb loss-related factors: time from limb loss, age at limb loss, limb loss reason, side and level of limb loss, unilateral/bilateral loss;
- prosthesis-related factors: type and number of used prostheses, prosthesis satisfaction related to appearance, comfort, control, reliability, glove durability, dexterity, donning/doffing, noise level, weight, cost of the prosthesis and of its maintenance, speed of opening and closing, grip strength, wrist control, ability to coordinate multiple joints, prosthesis related needs, wearing time, prosthesis usefulness, desired prosthesis improvement;
- prosthesis functionality: since the word “function” can be interpreted in different ways (i.e., to define the user ability to perform ADLs or to characterize the device performance, such as speed or grip strength), the terminology in the questionnaires should be better defined and explained also carrying out examples. In this paper the device performance is included in the prosthesis-related factors while grasping configurations, manipulation tasks and ADLs the user is able to perform, so as the physical effort needed to use the device, should be included in prosthesis functionality factors;
- rehabilitation factors: prosthesis training (if the user ever had prosthesis training, duration of the training, if he/she perceived advantages after training), follow-up. As outlined in Østlie et al. (2012), individualized prosthesis training should be mandatory in order to improve the prosthesis use.

In order to better compare the results obtained in the studies performed by different research groups, the introduction of a common evaluation scale regarding prosthetic usefulness, such as Likert scale, should be considered.

An in-depth analysis of bilateral prosthesis user needs and priorities should be carried out. Despite the analyzed studies typically involved both unilateral and bilateral users, the presented results are more related to unilateral users because more representative of the statistic sample. The data related to bilateral users require to be analyzed separately from unilateral data in order to correctly assess their feeling with prostheses. The unilateral users typically employ the prosthetic hand as assistive device of the intact limb meanwhile the bilateral users depend significantly more on the device. As pointed out in Østlie et al. (2012) the bilateral users pay more attention, in comparison with unilateral users, to activities related to personal hygiene, such as using a water tap, combing one's hair and blowing one's nose, that typically unilateral users perform with the intact limb.

## Conclusions and future perspectives

This work contributes to review the literature on the needs of upper limb prosthetic users and extracts the main requirements to be satisfied by the prosthetic systems in order to improve their acceptability and usefulness. The analysis has been focused on seven recent studies, Kyberd and Hill (2007), Biddiss et al. (2007), Jang et al. (2011), Pylatiuk et al. (2007), Østlie et al. (2012), Bouffard et al. (2012), and Lucchetti et al. (2015) which represent the most exhaustive ones in the literature and also include the results of previous studies, about level of satisfaction, needs and preferences of patients wearing upper limb prostheses.

In the proposed literature analysis special attention has been devoted to the comparative analysis of myoelectric, electric, body-powered and passive upper limb prostheses. In particular, for each study the type of prosthesis, level of limb loss, population, answered questions and percentage of response have been reported.

The thorough investigation of the patient needs reveals the requirements the prosthetic system of the future should meet. They can be summarized as follows:

1. To allow the execution of the daily life tasks more important to patients, ensuring the realization of the grasping and manipulation operations identified in Section Needs Analysis of Upper LIMB Prosthesis Users;
2. To integrate into the prosthesis a tactile sensorization system made of contact, sliding, temperature and force sensors;
3. To provide a control system able to command hand actuators independently managing position and force exerted by the fingers on the objects. The role of the visual feedback should be then lightened giving more importance to sensory feedback;
4. To increase the number of grasp types and to move toward manipulation tasks related to everyday life activities (feeding, dressing, cleanliness);
5. To increase the dexterity of the prosthesis enlarging the number of controlled degrees of freedom.

A possible, but not the only one, functional scheme of a prosthetic system (Figure 5) able to satisfy the emerged user wishes should be able to communicate in a bidirectional way with the PNS with both aims of controlling in a stable and dexterous way the hand prosthesis and of providing the amputee the tactile sensation related to the task she/he executes.

Furthermore, a list of factors necessary to assess user demands on prostheses has been identified.

In conclusion, despite the great progress made over the years in prosthetic field, this paper reveals the necessity to improve the hand control in order to assure tasks of daily living and to integrate the sensory feedback so as to restore the amputee sensations related to the interaction with the environment.

## Author contributions

FC and AC designed the paper, analyzed the literature and wrote the paper; RS, AD, AGC collaborated during the literature analysis; EG and LZ designed the paper and supervised the writing. All the authors read and approved the manuscript.

## Funding

This work was supported partly by the Italian Institute for Labour Accidents (INAIL) in the framework of the PPR 2 project (CUP: E58C13000990001), partly by the Italian Ministry of Instruction, University and Research in the framework of the PRIN HANDBOT project (CUP: B81J12002680008) and partly by the Italian Ministry of Health in the framework of the NEMESIS project (CUP: C81J12000380001).

### Conflict of interest statement

The authors declare that the research was conducted in the absence of any commercial or financial relationships that could be construed as a potential conflict of interest.

## Footnotes

^1^http://www.salute.gov.it/ricoveriOspedalieri/ric_informazioni/default.jsp

^2^http://www.nasdab.co.uk/

^3^COAPT Complete control, 2014 Available: http://www.coaptengineering.com/

^4^http://www.touchbionics.com/

^5^http://www.bebionic.com

^6^http://www.living-with-michelangelo.com/home/<
